# Red Flags, Prognostic Impact, and Management of Patients With Cardiac Amyloidosis and Aortic Valve Stenosis: A Systematic Review and Meta-Analysis

**DOI:** 10.3389/fmed.2022.858281

**Published:** 2022-03-09

**Authors:** Veronika A. Myasoedova, Maddalena Conte, Vincenza Valerio, Donato Moschetta, Ilaria Massaiu, Laura Petraglia, Dario Leosco, Paolo Poggio, Valentina Parisi

**Affiliations:** ^1^Centro Cardiologico Monzino Istituto di Ricovero e Cura a Carattere Scientifico (IRCCS), Milan, Italy; ^2^Dipartimento di Scienze Mediche Traslazionali, Università degli Studi di Napoli Federico II, Naples, Italy; ^3^Casa di Cura San Michele, Maddaloni, Italy; ^4^Dipartimento di Scienze farmacologiche e biomolecolari, Università degli Studi di Milano, Milano, Italy; ^5^Developmental Biology of the Immune System, Life and Medical Sciences Institute, University of Bonn, Bonn, Germany

**Keywords:** aortic stenosis (AS), cardiac amyloidosis (CA), outcome, surgical aortic valve replacement, transcatheter aortic valve implantation (TAVI)

## Abstract

**Background:**

Cardiac amyloidosis (CA) has been recently recognized as a condition frequently associated with aortic stenosis (AS). The aim of this study was to evaluate: the main characteristics of patients with AS with and without CA, the impact of CA on patients with AS mortality, and the effect of different treatment strategies on outcomes of patients with AS with concomitant CA.

**Materials and Methods:**

A detailed search related to CA in patients with AS and outcomes was conducted according to the Preferred Reporting Items for Systematic Reviews and Meta-Analyses (PRISMA) guidelines. Seventeen studies enrolling 1,988 subjects (1,658 AS alone and 330 AS with CA) were included in the qualitative and quantitative analysis of main patients with AS characteristics with and without CA, difference in mortality, and treatment strategy.

**Results:**

The prevalence of CA resulted in a mean of 15.4% and it was even higher in patients with AS over 80 years old (18.2%). Patients with the dual diagnosis were more often males, had lower body mass index (BMI), were more prone to have low flow, low gradient with reduced left ventricular ejection fraction AS phenotype, had higher E/A and E/e', and greater interventricular septum hypertrophy. Lower Sokolow–Lyon index, higher QRS duration, higher prevalence of right bundle branch block, higher levels of *N*-terminal pro-brain natriuretic peptide, and high-sensitivity troponin T were significantly associated with CA in patients with AS. Higher overall mortality in the 178 patients with AS + CA in comparison to 1,220 patients with AS alone was observed [odds ratio (OR) 2.25, *p* = 0.004]. Meta-regression analysis showed that younger age and diabetes were associated with overall mortality in patients with CS with CA (*Z*-value −3.0, *p* = 0.003 and *Z*-value 2.5, *p* = 0.013, respectively). Finally, patients who underwent surgical aortic valve replacement (SAVR) or transcatheter aortic valve implantation (TAVI) had a similar overall mortality risk, but lower than medication-treated only patients.

**Conclusion:**

Results from our meta-analysis suggest that several specific clinical, electrocardiographic, and echocardiographic features can be considered “red flags” of CA in patients with AS. CA negatively affects the outcome of patients with AS. Patients with concomitant CA and AS benefit from SAVR or TAVI.

## Introduction

In the geriatric population, calcific aortic stenosis (AS) is one of the most prevalent cardiovascular diseases. It affects 3% of the general population older than 65 years (1). AS is an age-related disease (2, 3) and, therefore, the number of affected patients will drastically increase in the coming decades due to the aging of the world population (4). Nowadays, the only therapeutic strategies available for AS are surgical aortic valve replacement (SAVR) or transcatheter aortic valve implantation (TAVI).

Cardiac amyloidosis (CA) is characterized by extracellular amyloid infiltration into the heart, in most cases of light chain (AL) or transthyretin (TTR) types. Acquired TTR amyloidosis (ATTRwt) is also called “senile amyloidosis” and is highly prevalent in the elderly (5).

The prevalence of CA in patients with AS is variable although consistent, ranging from 8 (6) to 16%, as reported in patients undergoing TAVI (7). The uncertainty about the prevalence of CA in the context of AS probably originates from the limited number of patients enrolled in most of the published studies. However, a different prevalence could be also explained by some specific patient's characteristics, in particular age and gender (6, 7). Two recent meta-analyses report a prevalence of CA in patient with AS of 9 and 14.4%, respectively (8, 9). Thus, the true prevalence of CA in patients with AS is still undefined and probably underestimated in some studies. A further increase in prevalence is expected in the next years, both due to the aging of the population and the improvement of the diagnostic algorithm (10), including multimodality imaging (11, 12).

The identification of “red flags” of CA in the context of AS is particularly challenging because the two diseases share common features. Some studies observed the clinical characteristics of patients with concomitant AS and CA and concluded that such patients show manifestations of more advanced disease, such as higher levels of *N*-terminal pro-brain natriuretic peptide (NT-proBNP), greater left ventricular (LV) hypertrophy, and advanced diastolic dysfunction (7, 8).

The outcome of patients with both the AS and CA is another important open question. There are conflicting reports on the prognostic significance of CA in patients with AS and on the potential impact of CA on aortic valve replacement benefits. Some studies attribute to CA an important role in AS prognosis (6, 13, 14), while other reports show that CA does not significantly worse AS outcome (15–17). It remains also to be clarified if there is a better therapeutic strategy in patients with both the AS and CA and if the presence of CA should be considered as an additive factor to be evaluated in the choice of AS treatment modality (SAVR or TAVI).

We performed a systematic review and meta-analysis to clarify: (1) the prevalence of CA in patients with AS; (2) the “red flags” of CA in the context of AS; (3) the impact of CA on patients with AS outcome; (4) the impact of aortic valve replacement in patients with concomitant CA and AS; and (5) if there is a treatment of choice (SAVR or TAVI) in patients with concomitant CA and AS.

## Materials and Methods

### Search Strategy

To perform a complete search and analysis, a detailed protocol for this review was prospectively developed, specifying objectives, criteria for study selection, outcomes, and statistical methods.

To identify all the available studies, a systematic search was evaluated in the electronic databases (PubMed, Web of Science, and Scopus). A detailed search relating to CA in patients with AS and outcomes was conducted according to the Preferred Reporting Items for Systematic Reviews and Meta-Analyses (PRISMA) guidelines (18). Following the search, terms were used in all the possible combinations: cardiac amyloidosis, aortic stenosis, aortic valve stenosis, outcome, adverse event, prognosis, risk stratification, and apical sparing. The last search was performed on December 29, 2021. The reference lists of all the retrieved articles were manually reviewed. Two independent authors (VAM and VP) analyzed each article and performed the data extraction independently. In case of disagreement, a third investigator was consulted (PP). Discrepancies were resolved by consensus.

### Data Extraction and Quality Assessment

According to the prespecified protocol, all the studies evaluating the prevalence and/or outcomes of concomitant CA and AS and the impact on cardiovascular risk factors were included. Case reports, case series not reporting data on prevalence and outcomes, reviews, and animal studies were excluded. We included in the analysis only studies on patients with AS with suspected or confirmed CA at echocardiography, cardiac magnetic resonance (CMR), and bone scintigraphy. In particular, we included two echocardiographic studies that identified patients with concomitant CA and severe AS with apical sparing pattern at LV strain analysis (19, 20). In each study, data regarding major clinical, demographic, echocardiographic, electrocardiographic variables, and prevalence of cardiovascular risk factors in patients with AS with and without CA were extracted. The quality analysis for each included study was performed accordingly to the Newcastle–Ottawa Scale (NOS). The result of the NOS quality assessment is given in Supplementary Table 1.

### Statistical Analysis and Risk of Bias Assessment

Statistical analysis was performed using Comprehensive Meta-analysis Version 3.3.070 (Biostat, Englewood, New Jersey, 2014). Differences among cases and controls in dichotomous variables were expressed as odds ratio (OR) with pertinent 95% CI and the differences in continuous variables were expressed as a standardized mean difference (SDM) and 95% CI. The overall effect was tested using *Z* scores and significance was set at *p* < 0.05. Statistical heterogeneity among studies was assessed with the chi-squared Cochran's *Q*-test and with *I*^2^ statistic, which measures the inconsistency across study results and describes the proportion of total variation in study estimates, that is due to heterogeneity rather than sampling error. In detail, *I*^2^ values of 0% indicate no heterogeneity, 25% low, 25–50% moderate, and 50% or more high heterogeneity (21).

Publication bias was assessed by Egger's test and represented graphically by funnel plots of the standard difference in means vs. the SE. Visual inspection of funnel plot asymmetry was performed to address for possible small-study effect and Egger's test was used to assess publication bias, over and above any subjective evaluation (22). The value *p* < 0.05 was considered as statistically significant. In order to be as conservative as possible, the random-effect method was used for all the analyses to consider the variability among the included studies (22). In the case of significant publication bias, Duval and Tweedie's trim and fill method was used to allow for the estimation of adjusted effect size (22).

### Meta-Regression Analyses

The differences in mortality among patients with AS with and without CA may be affected by clinical and demographic characteristics of patients included in different studies [mean age, diabetes, dyslipidemia, hypertension, body mass index (BMI), and coronary artery disease presence], echocardiographic characteristics (peak aortic jet velocity, mean aortic valve gradient, aortic valve area, LV ejection fraction, E/A ratio, E/e' ratio, interventricular septum, stroke volume index, and LV mass index), electrocardiographic characteristics (low voltage, Sokolow–Lyon Index, QRS duration, and right bundle branch block), and biochemical parameters. To assess the possible effect of such variables in explaining the different results observed across studies, meta-regression analyses after implementing a regression model with the mortality as dependent variables (*y*) and the variables mentioned above as independent variables (*x*) were performed (23).

## Results

**Figure 1:**
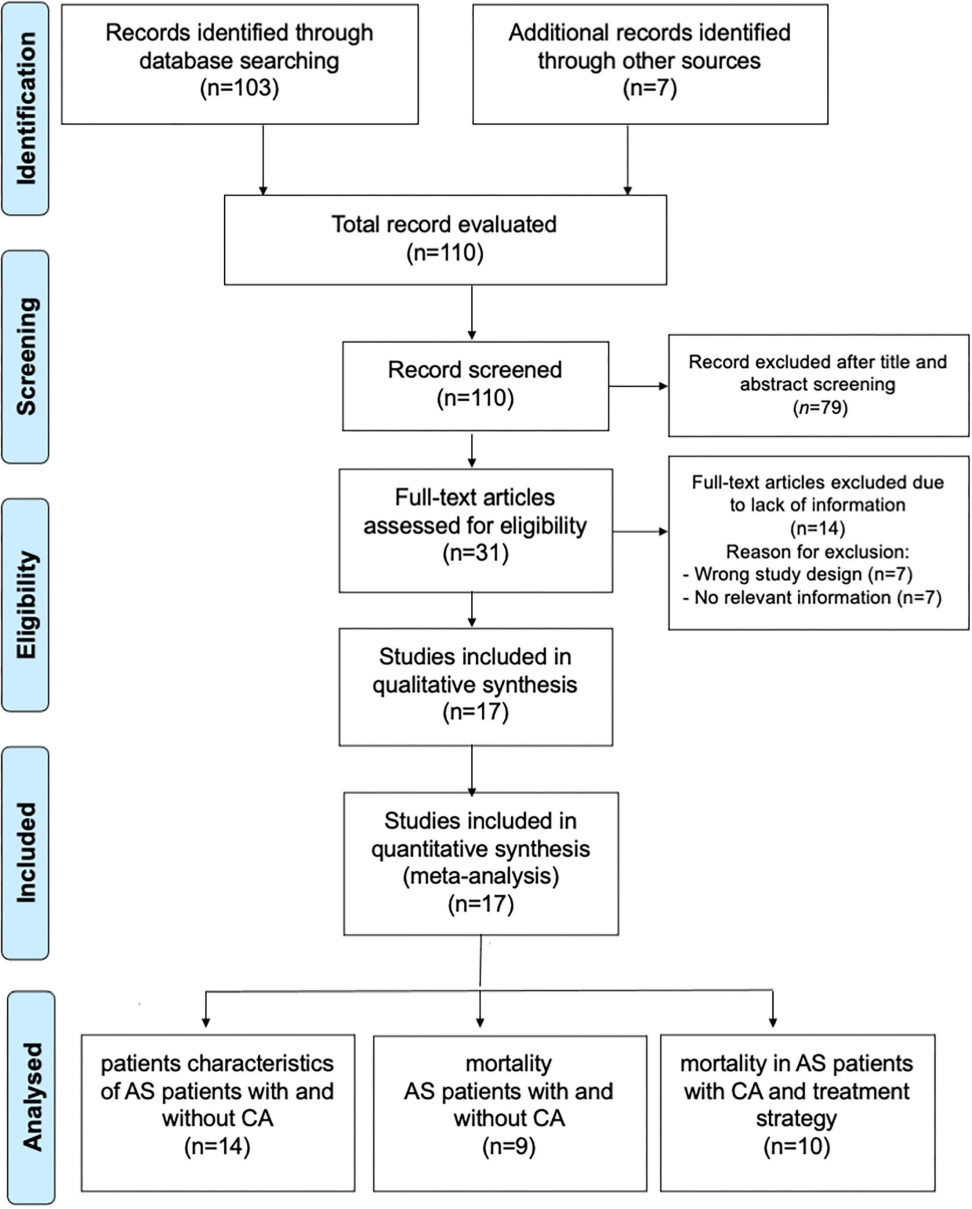
Prisma Flow Chart. The flow chart represents the number of studies evaluated according to PRISMA guidelines.

The search strategy identifies 110 articles (Figure 1). Duplicate results were excluded and after a screening of the titles and the abstracts, thirty-one articles were selected for full-text evaluation. The revision of full-length articles allowed the exclusion of fourteen studies due to wrong study design or irrelevant information in their content. Thus, seventeen studies (6, 7, 13–17, 19, 20, 24–31), enrolling 1,988 subjects (1,658 AS alone and 330 AS with CA), were included in the qualitative and quantitative analyses of main patients with AS characteristics with and without CA (14 studies), difference in mortality (9 studies), and treatment strategy (10 studies).

### Prevalence of CA and Characteristics of AS Patients With and Without CA

**Figure 2:**
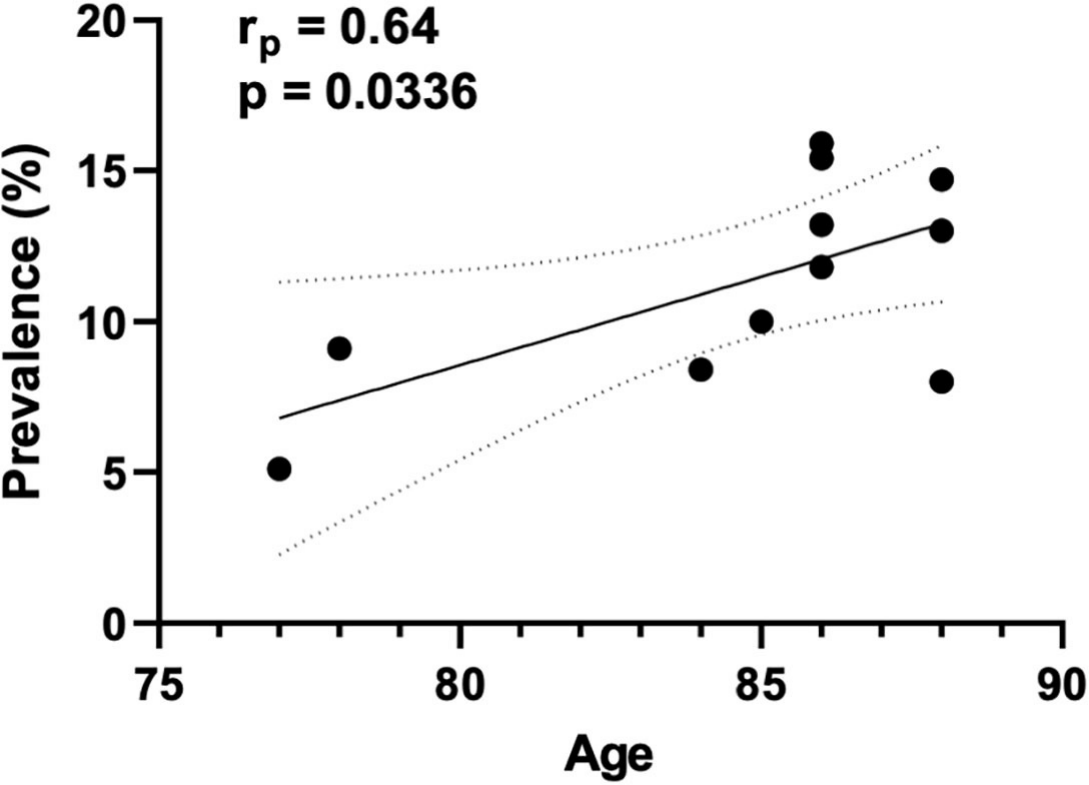
Correlation between the prevalence of CA and age. The correlation plot indicates the results of 11 studies. The abscissa (x axis) represents the mean age of patients with both conditions (CA and AS), while the ordinate (y axis) represent the prevalence of CA in each included study.

The prevalence of CA was accessed in 14 studies (6, 7, 13–17, 19, 20, 25, 28–31), including 1,934 subjects (276 with concomitant CA and AS), resulting in a mean of 15.4%. Our results suggest that the prevalence of CA is lower in patients with AS under 80 years old compared with patients over 80 years old (7.1 vs. 18.2%, respectively). Of note, we found a positive correlation between the prevalence of CA in each study and the age of these patients (Figure 2).

Clinical and demographic characteristics as well as cardiovascular risk factors associated with CA prevalence in patients with AS were reported in 12 studies (6, 7, 13, 15–17, 19, 20, 28–31), including 1,772 subjects (1,506 AS alone and 266 AS with CA) (Table 1; Supplementary Figures 1, 2).

**Table 1:** Characteristics of patients with AS with and without CA.

**Variable**	**AS + CA**	***n***	**AS**	**n**	**Effect size**	***P*-value**
Age, years	85.4 ± 5.5	259	82.4 ± 8.8	1,441	0.38 (0.24; 0.51)	**<0.001**
Male, *n* (%)	180 (67.7)	266	784 (52.1)	1,506	2.01 (1.13; 3.56)	**0.017**
BMI, kg/m^2^	25.6 ± 3.9	181	26.1 ± 4.8	893	−0.32 (−0.51; −0.12)	**0.002**
Hypertension, *n* (%)	212 (80.9)	262	1,170 (79.6)	1,470	1.12 (0.71; 1.77)	0.638
Diabetes, *n* (%)	48 (22.7)	211	297 (25.5)	1,166	0.95 (0.59; 1.51)	0.811
Dyslipidemia, *n* (%)	66 (43.7)	151	332 (47.8)	674	0.80 (0.54; 1.18)	0.261
CAD, *n* (%)	108 (49.1)	220	555 (46.1)	1,203	1.15 (0.75; 1.77)	0.530
Stage D1	63 (54.8)	115	605 (72.2)	838	0.45 (0.30;0.68)	**<0.001**
Stage D2	37 (29.8)	124	171 (18.2)	942	2.26 (1.44; 3.54)	**<0.001**
Stage D3	24 (20.9)	115	118 (14.1)	838	1.77 (0.86; 3.61)	0.119
AV mean gradient, mmHg	39.5 ± 15.6	259	42.9 ± 13.6	1,441	−0.33 (−0.54; 0.11)	**0.003**
AV peak velocity, cm/s	3.9 ± 0.8	195	4.2 ± 0.6	1,112	−0.41 (−0.68; −0.14)	**0.003**
AV Area, cm^2^	0.7 ± 0.23	259	0.7 ± 0.19	1,441	−0.006 (−0.29; 0.28)	0.964
E/A ratio	1.7 ± 1.1	141	0.9 ± 07	930	2.26 (1.3; 4.7)	**<0.001**
E/e' ratio	21.8 ± 11.0	129	17.5 ± 8.6	836	0.48 (0.29; 0.67)	**<0.001**
LVEF, %	53 ± 14	262	58 ± 14	1,470	−0.40 (−0.59; −0.20)	**<0.001**
IVS, mm	14.5 ± 3.3	224	12.9 ± 2.5	1,456	0.67 (0.46; 0.88)	**<0.001**
SVi, mL/m^2^	30 ± 10	224	38 ± 15	1,427	−0.52 (−0.68; −0.35)	**<0.001**
LV Mass index, g/m^2^	139 ± 42	220	117 ± 33	1,391	0.75 (0.45; 1.05)	**<0.001**
Low voltage, *n* (%)	9 (4.2)	214	52 (4.0)	1,316	1.59 (0.77; 3.27)	0.209
Sokolow-Lyon Index, mV	1.9 ± 0.7	154	2.4 ± 0.9	1,060	−1.3 (−2.02; 0.54)	**0.001**
QRS duration, ms	128 ± 27	184	102 ± 25	1,266	0.36 (0.05; 0.66)	**0.002**
RBBB, *n* (%)	48 (30.0)	160	121 (10.7)	1,134	3.55 (2.32; 5.41)	**<0.001**
NT-proBNP, ng/l	3,338 ± 3,362	160	1,558 ± 2,273	978	0.76 (0.43; 1.09)	**<0.001**
Hs-TnT, ng/l	41.2 ± 34.4	106	23.4 ± 17.8	830	0.93 (0.72; 1.13)	**<0.001**

*AS, aortic stenosis; CA, cardiac amyloidosis; BMI, body mass index; CAD, coronary artery disease; D1, high gradient; D2, low-flow, low-gradient with reduced LVEF; D3, low-flow, low-gradient with normal LVEF; AV, aortic valve; LV, left ventricular; LVEF, left ventricular ejection fraction; IVS, interventricular septum; SVi, stroke volume index; RBBB, right bundle branch block; NT-proBNP, N-terminal pro-brain natriuretic peptide; Hs-TnT, high-sensitivity troponin T. statistically significative values (p < 0.05) are reported in bold*.

Patients with concomitant CA and AS were more often males, 68 vs. 52%, with a corresponding odds ratio (OR) of 2.01 (95% CI: 1.13, 3.56; *p* < 0.0001) and had lower BMI, SDM of −0.32 (95% CI: −0.51; −0.12, *p* = 0.002). Analysis of echocardiographic parameters found that patients with AS and CA were more prone to have low flow, low gradient with reduced LV ejection fraction (LVEF), OR of 2.26 (95% CI: 1.44; 3.54, *p* < 0.001), lower aortic valve mean gradient SDM of −0.33 (95% CI: −0.54; 0.11, *p* = 0.003), and higher E/A and E/e' ratios SDM of 2.26 (95% CI: 1.3; 4.7, *p* < 0.001) and 0.48 (95% CI: 0.29; 0.67, *p* < 0.001), respectively. Interventricular septum (IVS) was thicker in patients with dual diagnosis (SDM 0.67, 95% CI: 0.46; 0.88, *p* < 0.001), while stroke volume index (SVi) was higher in patients with AS alone (SDM −0.52, 95% CI: −0.68; −0.35, *p* < 0.001). Electrocardiographic parameters significantly associated with AS and CA were: lower Sokolow–Lyon Index (SDM −1.3, 95% CI: −2.02; 0.54, *p* < 0.001); higher QRS duration (SDM 0.36, 95% CI: 0.05; 0.66, *p* = 0.002); and higher prevalence of right bundle branch block (RBBB) (OR 3.55, 95% CI: 2.32; 5.41, *p* < 0.001). Finally, higher levels of both the NT-proBNP and high-sensitivity troponin T (Hs-TnT) were significantly associated with AS and CA in comparison with the patients with AS alone, SDM of 0.76 (95% CI: 0.43; 1.09, *p* < 0.001) and 0.93 (95% CI: 0.72; 1.13, *p* < 0.001), respectively. The heterogeneity of all the analyzed variables is given in Supplementary Table 2. In particular, data of male sex, aortic valve mean gradient, E/A ratio, IVS, Sokolow–Lyon Index, NT-proBNP, and Hs-TnT showed significant heterogeneity, while no heterogeneity was observed for BMI, presence of low flow, low gradient with reduced LVEF, E/e' ratio, and QRS duration.

### Publication Bias

Funnel plots of effect size vs. SE for all the performed analyses were rather symmetrical and Egger's test showed the absence of publication bias, except for three variables, such as age, E/A ratio, and LV mass index. These variables showed an asymmetric distribution and Egger's test confirmed the presence of a significant publication bias (Egger's *p* = 0.023, *p* = 0.019, and *p* = 0.045, respectively; Supplementary Table 3). However, the Duval and Tweedie's trim and fill analysis showed that, after adjusting for publication bias, results were confirmed (age, SDM of 0.33, 95% CI: 0.13, 0.53; E/A ratio, SDM of 1.4, 95% CI: 0.35, 2.44; LV mass index 0.49, 95% CI: 0.15, 0.82; Supplementary Figure 3).

### Increased Mortality in Patients With AS and Concomitant CA

**Figure 3:**
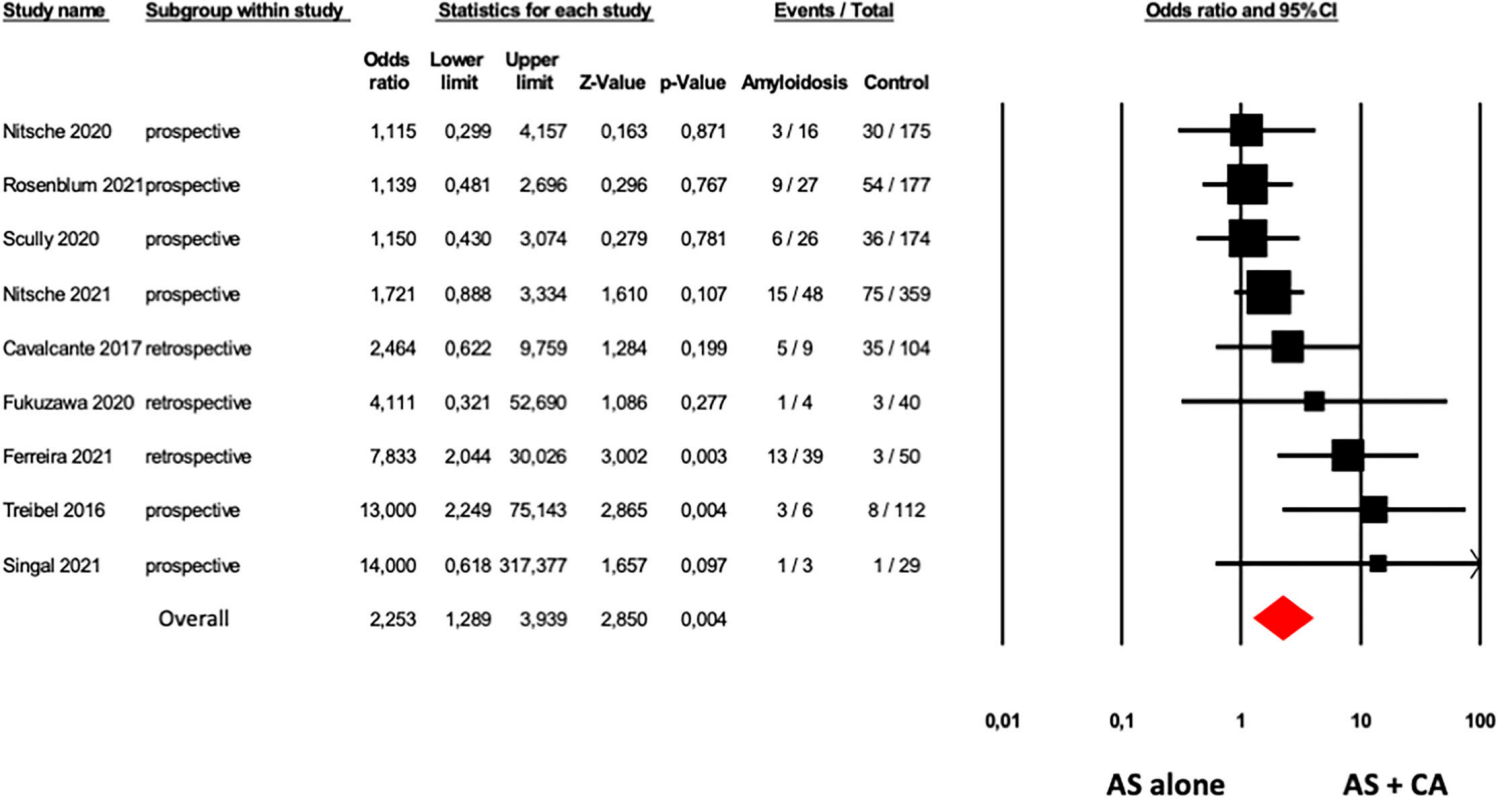
Forest plots of overall mortality in AS patients with and without CA. Overall mortality was evaluated with the difference in Odds Ratio (OR) between AS patients with and without CA. The diamond represents the estimated overall effect, while the squares represent each study with 95%CI.

The presence of CA was associated with an increased mortality rate in patients with AS. Nine studies (6, 13–17, 19, 25, 31) showed higher overall mortality in the 178 patients with AS with CA in comparison with 1,220 patients with AS alone with an OR of 2.25 (95% CI: 1.23–3.94, *p* = 0.004), with a moderate heterogeneity among studies (*I*^2^: 43% *p* = 0.082; Figure 3).

Funnel plots of effect size vs. SE for overall mortality in patients with AS with and without CA showed an asymmetric distribution and Egger's test confirmed the presence of a significant publication bias (Egger's *p* = 0.048, Supplementary Figure 4). After adjusting for publication bias (Duval and Tweedie's trim and fill analysis), results were consistently confirmed with an OR of 1.85 (95% CI: 1.01, 3.40).

Sensitivity analysis showed that, excluding the study of Ferreira et al. (19), due to different methods of CA evaluation, considering eight studies (6, 13–17, 25, 31) including 139 patients with AS with CA and 1,170 patients with AS without CA, the results were confirmed with an OR of 1.83 (95% CI: 1.11, 3.03, *p* = 0.019), without low heterogeneity among studies (*I*^2^: 26% *p* = 0.219).

### Meta-Regression Analysis

**Figure 4:**
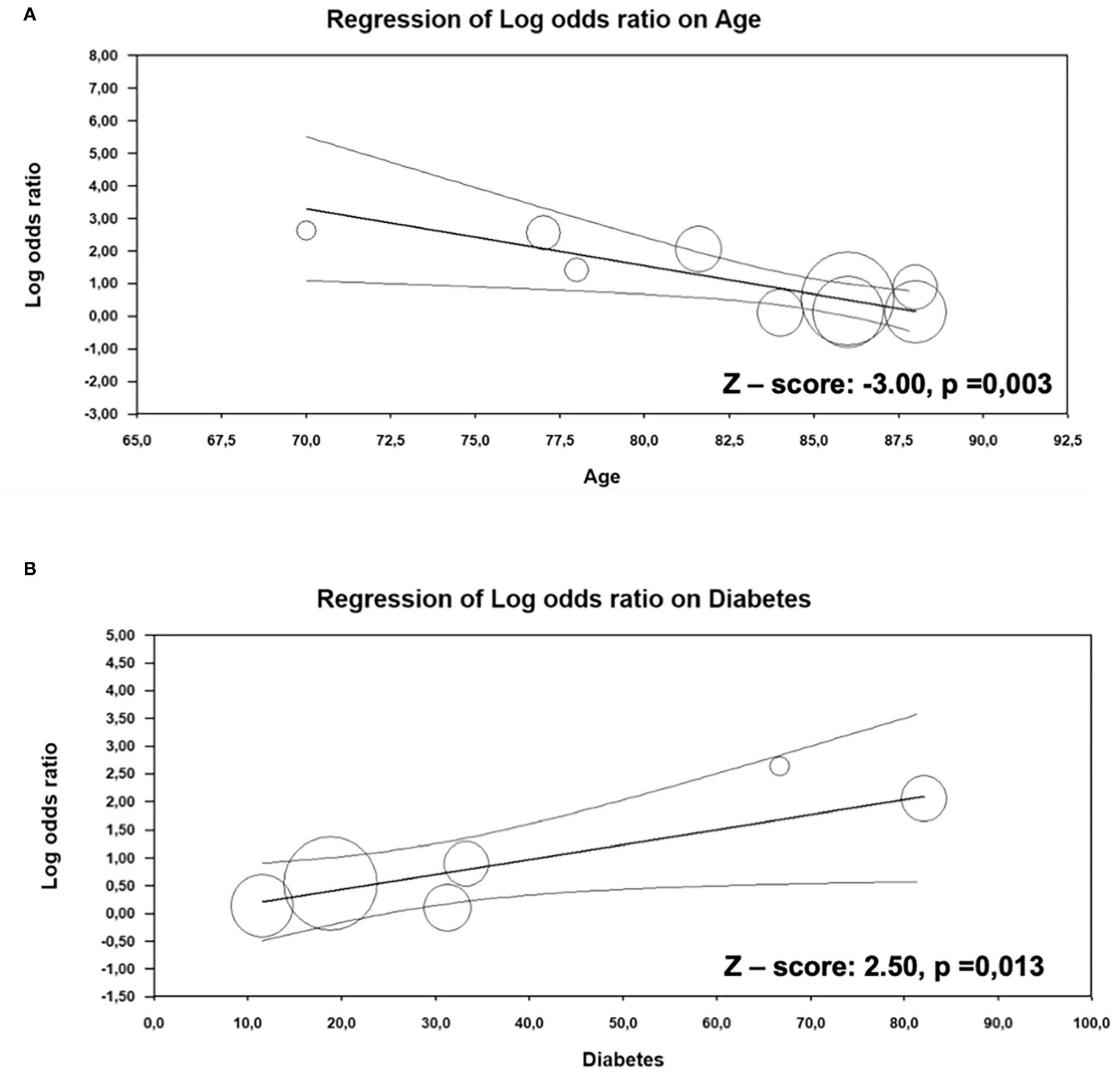
Meta-regression analysis. Impact of age **(A)** and diabetes **(B)** on the difference in overall mortality between AS patients with and without CA.

For the evaluation of the impact of major clinical and demographic characteristics on the difference in overall mortality between patients with AS alone and with CA, meta-regression models were performed (Supplementary Table 4). Our results showed that with increasing age, the difference in overall mortality rate between patients with AS with and without CA declines, suggesting that the presence of CA in younger age in patients with AS could lead to higher mortality risk (*Z*-value: −3.0; *p* = 0.003, Figure 4A). In addition, diabetes was positively associated with overall mortality in patients with AS with CA (*Z*-value: 2.5; *p* = 0.013, Figure 4B).

### Different Treatment Strategies and Overall Mortality in Patients With Concomitant CA and AS

**Figure 5:**
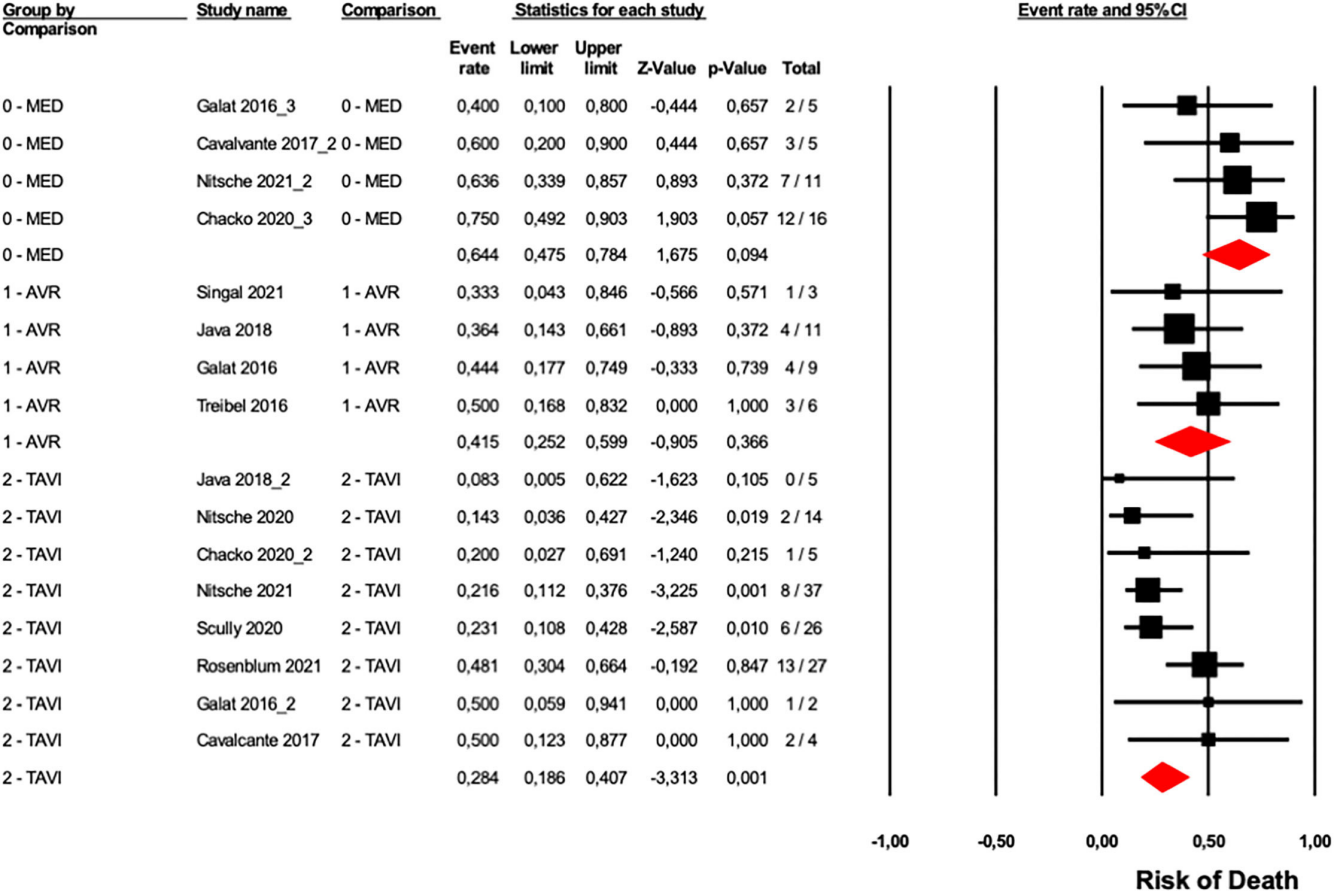
Forest plots of overall mortality in a different type of treatment in AS patients with CA. Overall mortality was evaluated with the difference in event rate between different types of AS patients treatments with and without CA. The diamond represents the estimated overall effect, while the squares represent each study with 95%CI.

To evaluate the role of CA in patients with AS who underwent a different type of medical intervention, we analyzed data from ten studies (6, 13–17, 24, 26, 27, 31), four studies reported data on medical/pharmacological treatment (6, 13, 24, 26), four studies reported data on SAVR (14, 26, 27, 31), and eight studies included data regarding TAVI (6, 13, 15–17, 24, 26, 27). Results of our analysis suggested a significant difference in overall mortality between the three groups (*p* = 0.002, Figure 5). As expected, patients who underwent SAVR or TAVI showed a lower risk of overall mortality compared with the patients pharmacologically treated only. However, no difference was observed between patients treated with SAVR *vs*. TAVI (*p* = 0.217).

Funnel plots of effect size vs. SE for all the performed analyses were rather symmetrical and Egger's test showed the absence of publication bias (Supplementary Table 5).

## Discussion

In the present systematic review and meta-analysis, we report that: (1) in elderly patients with AS, the prevalence of CA increases with age; (2) specific clinical, electrocardiographic, and echocardiographic features can be considered “red flags” of CA in patients with AS; (3) CA negatively affects the outcome of patients with AS; (4) patients with concomitant CA and AS benefit from aortic valve replacement; and (5) in patients with concomitant CA and AS, there is not a treatment of choice.

The prevalence of CA in patients with AS is highly variable in literature. In the analyzed studies, it ranges from 8 to 16% (6, 7). This variability could be in part explained by the use of different imaging diagnostic techniques. However, the variability of CA prevalence in patients with AS remains high even among studies based on the same diagnostic method (7, 15). Another possible explanation could be the limited number of patients enrolled in most of the studies, but also two recent meta-analysis report a different prevalence of CA in patients with AS ranging from 9 to 14.4% (8, 9). Thus, it is plausible that specific patient's characteristics of the populations included in the studies could explain such differences. In this study, the overall prevalence of CA in patients with AS was of 15.4%. Although all the patients included were elderly, we observed a higher prevalence of CA in those with more than 80 years old (18.2 vs. 7.1%). Among the analyzed demographic and clinical variables, age, male—gender, and BMI were significantly associated with the presence of CA in patients with AS. Cavalcante et al. (6) described that, in a population of 113 patients with severe AS, the prevalence of CA increased from 8 to 25% when only older (≥80 years) and male patients were considered. Thus, we can argue that the more “red flags” are present in a selected population, the higher the CA prevalence will be. In the present systematic review and meta-analyses, by pooling data of 226 patients (6, 7, 13, 15–17, 19, 20, 28–31), we identified the factors associated to CA in patients with AS. We observed that patients with both the CA and AS, together with the described demographic and clinical variables, are characterized by a low-flow low-gradient AS phenotype, worse diastolic LV function, greater LV hypertrophy, higher NT-proBNP and Hs-TnT, and specific ECG features (RBBB, reduced Sokolow–Lyon Index, and increased QRS duration). A recent expert consensus of the European Society of Cardiology (10) suggests that the presence of LV hypertrophy (LV wall thickness ≥12 mm) is sufficient in patients with AS to arise the suspicion of CA and proceed in the diagnostic algorithm based on bone scintigraphy/CMR coupled to assessment for monoclonal proteins. However, in clinical practice the systematic use of bone scintigraphy and/or CMR could not be feasible in all the patients, both for logistic and economic reasons (32). Thus, the identification and validation of “red flags” scores for CA in patients with AS is probably the most important challenge. Nitsche et al. (13) proposed the remodeling, age, injury, systemic, and electrical (RAISE) score to standardize the CA assessment in patients with AS. These authors assigned a different weight to each factor and suggested that patients with a score of >2 points necessitate of further screening by bone scintigraphy and light-chain assessment. Given its impact on AS prognosis, the identification of CA in patients with AS is particularly important. Our results confirm the evidence of previous meta-analyses (8, 9) on the adverse outcome of patients with both the pathologies. We analyzed data from 9 studies (6, 13–17, 19, 25, 31) with an overall population of 1,398 patients, 178 affected by both the AS and CA. At a mean follow-up of 19 months, 245 (21%) and 56 (32%) patients died in the lone AS and CA-AS pooled study groups, respectively. The negative impact of CA on patients with AS was also confirmed when data were adjusted for publication bias. We confirmed that CA is associated to a worse prognosis in patients with AS even after a sensitivity analysis without the study including patients with only a probability of CA, based on echocardiographic strain analysis (19). Interestingly, the negative effect of CA on AS outcome appears to be reduced in older subjects, thus suggesting the importance of CA assessment especially in patients with AS younger than 80 years old. On the contrary, the presence of diabetes further increases the mortality risk of patients with both the CA and AS. Although the negative impact of CA on patient's outcome, this study confirms the benefits of aortic valve replacement with respect to medical therapy even in patients with both the diseases. The benefit of aortic valve replacement has been questioned by some authors that, in a small population, reported that patients with CA and AS died at the same rate as those with CA alone, despite some having undergone SAVR (33). On contrast, other authors recently reported that TAVI significantly improves the prognosis of patients with both the AS and CA, with a similar survival rate to patients with AS alone (15, 17).

An important open question is whether CA should be considered an additive factor able to influence the choice of treatment modality (SAVR or TAVI) in a single patient. Conflicting evidence is reported in the literature on possible postprocedural complications in patients with both the CA and AS. Some authors suggest a high risk for TAVI because of operative and postoperative complications, including atrioventricular blocks with need for permanent pacemaker implantation and risk of LV rupture (33–35). Java et al. reported possible complications even after SAVR, such as postoperative tamponade and low-output syndrome (27). In this study, we included 10 studies with an overall population of 120 patients referred to TAVI and 29 patients to SAVR. We compared the outcome of patients undergone TAVI and SAVR and we observed that the two treatments are similar in term of survival benefits. Indeed, as patients with CA are elderly and with more advanced disease, it is plausible that the evaluation of each single case by the heart team frequently favors the choice of TAVI. In the analyzed data, far fewer patients were referred to SAVR (only 29), thus indicating that, in clinical practice, clinicians often prefer TAVI in patients with both the CA and AS. It is important to underline that we could only analyze the differences of the two treatment modalities on mortality, without considering the benefits in terms of symptoms, heart failure progression, and patients' functional capacity recovery, all the items which could make the difference among the two treatment strategies. Finally, further studies are required to establish the benefits of concomitant aortic valve replacement and tafamidis in patients with AS and TTR amyloidosis.

### Study Limitations

This study has some potential limitations. In the analyzed publications, the diagnosis of CA was made by different imaging techniques; however, data were confirmed even without studies with uncertain CA diagnoses. Systemic red flags of CA, such as carpal tunnel syndrome, were not included in the analysis due to a lack of relevant data on concomitant carpal tunnel syndrome and CA in patients with AS. In articles evaluating outcomes, also patients with moderate AS were included, leading to an underestimation of overall mortality in particular of patients with AS referred to medical treatment. The small sample size in the different treatment strategies could explain the slightly decreased risk of overall mortality of patients with SAVR compared to pharmacologically treated ones. Finally, the results of this study were focused on overall mortality; however, the assessment of cardiovascular mortality, rehospitalization, and functional status could improve our understanding of CA role in AS context.

## Conclusion

The prevalence of CA in AS is consistent and increases with age. Patients with concomitant CA and AS are characterized by advanced age, male sex, lower BMI, and features of more advanced disease. The presence of CA confers a worse prognosis to patients with AS; however, the benefits of aortic valve replacement remain significant even in presence of both the diseases. Based on the analyzed studies, there is not a treatment of choice between SAVR and TAVI, but due to the low number of patients who undergo SAVR in clinical practice, randomized further studies are required to better define this issue. There are currently no data on the cumulative benefit of aortic valve replacement and tafamidis in patients with concomitant CA and AS.

## Data Availability Statement

The original contributions presented in the study are included in the article/Supplementary Material, further inquiries can be directed to the corresponding author/s.

## Author Contributions

VM and MC conceived of the presented manuscript. VM, PP, and VP analyzed each article and performed the data extraction independently. VV, DM, and IM draft the method and result section with the input of VM and PP. LP and DL draft the introduction and discussion section with the input of MC and VP. All authors discussed the results and contributed to the final manuscript.

## Funding

This study was supported by the Italian Ministry of Health funds (Ricerca Finalizzata: GR-2019-12370560) and by the Fondazione Gigi e Pupa Ferrari ONLUS (FPF-14).

## Conflict of Interest

The authors declare that the research was conducted in the absence of any commercial or financial relationships that could be construed as a potential conflict of interest.

## Publisher's Note

All claims expressed in this article are solely those of the authors and do not necessarily represent those of their affiliated organizations, or those of the publisher, the editors and the reviewers. Any product that may be evaluated in this article, or claim that may be made by its manufacturer, is not guaranteed or endorsed by the publisher.
