# Supplementary material for: Red Flags, Prognostic Impact, and Management of Patients With Cardiac Amyloidosis and Aortic Valve Stenosis: A Systematic Review and Meta-Analysis
Source: Front Med (Lausanne). 2022 Mar 9;9:858281. doi: 10.3389/fmed.2022.858281 (PMC8959832; doi:10.3389/fmed.2022.858281)
Supplement: Supplementary file 1 [file Data_Sheet_1.docx]

**SUPPLEMENTAL TABLE AND FIGURES**

**Red Flags, Prognostic Impact, and Management of Patients with Cardiac Amyloidosis and Aortic Valve Stenosis: A Systematic Review and Meta-analysis**

Myasoedova VA^1^, Conte M^2.3^, Valerio V^1^, Moschetta D^1.4^, Massaiu I^1.5^, Petraglia L^2^, Leosco D^2^, Poggio P^1^ and Parisi V^2^

*^1^ Centro Cardiologico Monzino IRCCS,20138 Milan Italy*

*^2^ Università degli Studi di Napoli Federico II, 80131, Napoli, Italy.*

*^3^ Casa di Cura San Michele, Maddaloni, Italy,*

*^4^ Università degli Studi di Milano, Milano, Italy,*

*^5^ Developmental Biology of the Immune System,Life and Medical Sciences Institute, University of Bonn, Bonn, Germany*

Running title: Amyloidosis and Aortic Valve Stenosis

^*^ These Authors equally contributed to the present work.

^#^ To whom correspondence should be addressed:

Valentina Parisi, MD PhD

Dipartimento di Scienze Mediche Traslazionali

Università degli Studi di Napoli Federico II

80131, Napoli, Italy.

Email: [parisi.valentina@tiscali.it](mailto:parisi.valentina@tiscali.it)

**Supplemental Table S1. Quality assessment with Newcastle-Ottawa scale of the included studies.**

| **Author, year** | **SELECTION** | | | | **COMPARABILITY** | **OUTCOME** | | |  |
| --- | --- | --- | --- | --- | --- | --- | --- | --- | --- |
|  | **Representativeness of the Exposed Cohort** | **Selection of the Non-Exposed Cohort** | **Ascertainment of Exposure** | **Demonstration That Outcome of Interest Was Not Present at Start of Study** |  | **Assessment of Outcome** | **Enough Follow-Up Long for Outcomes to Occur** | **Adequacy of Follow Up of Cohorts** | **Quality** |
| Castano *et al*., 2017 | * | * | * | - | * | * | - | - | **5** |
| Cavalcante *et al*., 2017 | * | * | * | - | * | * | * | * | **7** |
| Nitsche *et al*., 2020 | * | * | * | - | ** | * | * | * | **8** |
| Scully *et al*., 2020 | * | * | * | - | ** | * | * | * | **8** |
| Scully 2020_2 *et al*., | * | * | * | - | * | * | - | - | **5** |
| Saito *et al*., 2020 | * | * | * | - | ** | * | - | - | **6** |
| Rosenblum *et al*., 2021 | * | * | * | - | * | * | * | * | **7** |
| Nitsche *et al*., 2021 | * | * | * | - | ** | * | * | * | **8** |
| Ferreira *et al*., 2021 | * | * | * | - | * | * | * | * | **7** |
| Singal *et al*., 2021 | * | * | * | - | ** | * | * | * | **8** |
| Narotsky *et al*., 2015 | * | * | * | - | * | * | - |  | **5** |
| Robin *et al*., 2021 | * | * | * | - | ** | * | - | - | **6** |
| Treibel *et al*., 2016 | * | * | * | - | * | * | * | * | **7** |
| Fukuzawa *et al*., 2020 | * | * | * | - | * | * | * | * | **7** |
| Galat *et al*., 2016 | * | * | * | - | * | * | * | * | **7** |
| Sperry *et al*., 2016 | * | * | * | - | * | * | * | * | **7** |
| Java *et al*.,  2018 | * | * | * | - | * | * | * | * | **7** |
| Ali Ambreen *et al*., 2020 | * | * | * | - | * | * | * | * | **7** |
| Chacko *et al*., 2020 | * | * | * | - | * | * | * | * | **7** |

**Supplemental Table S2. Statistical heterogeneity among studies**

| **Variable** | **Q - Value** | **I - Squared** | **P - Value** |
| --- | --- | --- | --- |
| **Age, years** | 14.4 | 37.3 | 0.110 |
| **Male, n (%)** | 26.2 | 61.8 | **0.003** |
| **BMI, kg/m²** | 6.7 | 25.3 | 0.244 |
| **Hypertension, n (%)** | 14.9 | 33.1 | 0.134 |
| **Diabetes, n (%)** | 11.3 | 29.5 | 0.183 |
| **Dyslipidemia, n (%)** | 4.6 | 0 | 0.469 |
| **CAD, n (%)** | 13.6 | 41.3 | 0.092 |
| **Stage D1** | 1.8 | 0 | 0.617 |
| **Stage D2** | 3.5 | 0 | 0.478 |
| **Stage D3** | 4.8 | 37.4 | 0.188 |
| **AV mean gradient, mmHg** | 20.9 | 57.0 | **0.013** |
| **AV peak velocity, cm/s** | 17.5 | 65.8 | **0.007** |
| **Aortic Valve Area, cm^2^** | 36.7 | 75.5 | **<0.001** |
| **E/A ratio** | 82.5 | 95.2 | **<0.001** |
| **E/è ratio** | 6.1 | 1.4 | 0.414 |
| **LVEF, %** | 19.6 | 49.1 | **0.033** |
| **IVS, mm** | 19.2 | 47.8 | **0.038** |
| **SVi, mL/m^2^** | 11.5 | 21.5 | 0.245 |
| **LV Mass index, g/m^2^** | 31.1 | 74.3 | **<0.001** |
| **Low voltage, n (%)** | 5.2 | 0 | 0.741 |
| **Sokolow-Lyon Index, mV** | 88.8 | 93.8 | **<0.001** |
| **QRS duration, ms** | 23.6 | 70.3 | **0.001** |
| **RBBB, n (%)** | 6.6 | 9.0 | 0.360 |
| **NT-proBNP, ng/l** | 16.7 | 70.1 | **0.005** |
| **HsTnT, ng/l** | 1.8 | 0 | **0.776** |

AS: aortic stenosis; AV: aortic valve; BMI: body mass index; CA: cardiac amyloidosis; CAD coronary artery disease; D1: high gradient; D2: low-flow, low-gradient with reduced LVEF; D3: low-flow, low-gradient with normal LVEF; LV: left ventricular; IVS: intraventricular septum; LVEF: left ventricular ejection fraction; RBBB: right bundle branch block; SVi: stroke volume index

**Supplementary Table S3. Publication bias Egger’s regression intercept test.**

| **Variable** | **P - Value** |
| --- | --- |
| **Age, years** | **0.023** |
| **Male, n (%)** | 0.284 |
| **BMI, kg/m²** | 0.227 |
| **Hypertension, n (%)** | 0.357 |
| **Diabetes, n (%)** | 0.794 |
| **Dyslipidemia, n (%)** | 0.816 |
| **CAD, n (%)** | 0.267 |
| **Stage D1** | 0.424 |
| **Stage D2** | 0.938 |
| **Stage D3** | 0.553 |
| **AV mean gradient, mmHg** | 0.606 |
| **AV peak velocity, cm/s** | 0.970 |
| **Aortic Valve Area, cm^2^** | 0.924 |
| **E/A ratio** | **0.019** |
| **E/è ratio** | 0.399 |
| **LVEF, %** | 0.071 |
| **IVS, mm** | 0.116 |
| **SVi, mL/m^2^** | 0.106 |
| **LV Mass index, g/m^2^** | **0.045** |
| **Low voltage, n (%)** | 0.775 |
| **Sokolow-Lyon Index, mV** | 0.193 |
| **QRS duration, ms** | 0.395 |
| **RBBB, n (%)** | 0.080 |
| **NT-proBNP, ng/l** | 0.954 |
| **HsTnT, ng/l** | 0.912 |

AS: aortic stenosis; AV: aortic valve; BMI: body mass index; CA: cardiac amyloidosis; CAD coronary artery disease; D1: high gradient; D2: low-flow, low-gradient with reduced LVEF; D3: low-flow, low-gradient with normal LVEF; LV: left ventricular; IVS: intraventricular septum; LVEF: left ventricular ejection fraction; RBBB: right bundle branch block; SVi: stroke volume index

**Supplementary Table S4. Meta-regressions**

| **Variable** | **Z - Value** | **P - Value** |
| --- | --- | --- |
| **Age, years** | **-3.0** | **0.003** |
| **Male, n (%)** | -0.55 | 0.554 |
| **Hypertension, n (%)** | -1.24 | 0.216 |
| **Diabetes, n (%)** | 2.50 | **0.013** |
| **CAD, n (%)** | -0.02 | 0.986 |
| **Stage D2** | 0.77 | 0.440 |
| **AV mean gradient, mmHg** | 2.15 | **0.032** |
| **Aortic Valve Area, cm^2^** | -1.20 | 0.230 |
| **E/è ratio** | 0.09 | 0.929 |
| **LVEF, %** | 0.86 | 0.390 |
| **IVS, mm** | 1.12 | 0.263 |
| **SVi, mL/m^2^** | -0.18 | 0.854 |
| **LV Mass index, g/m^2^** | -0.38 | 0.705 |
| **Low voltage, n (%)** | 0.37 | 0.711 |
| **QRS duration, ms** | -1.25 | 0.213 |
| **RBBB, n (%)** | -1.29 | 0.197 |
| **HsTnT, ng/l** | -1.33 | 0.183 |

AS: aortic stenosis; AV: aortic valve; BMI: body mass index; CA: cardiac amyloidosis; CAD coronary artery disease; D1: high gradient; D2: low-flow, low-gradient with reduced LVEF; D3: low-flow, low-gradient with normal LVEF; LV: left ventricular; IVS: intraventricular septum; LVEF: left ventricular ejection fraction; RBBB: right bundle branch block; SVi: stroke volume index

**Supplementary Table S5. Publication bias Egger’s regression intercept test.**

| **Variable** | **P - Value** |
| --- | --- |
| **Medical/pharmacological treatment** | 0.167 |
| **Aortic Valve Replacement** | 0.898 |
| **Transcatheter Aortic Valve Implantation** | 0.630 |

**Supplementary Figure S1. Forrest plots of dichotomous variables**

**Supplementary Figure S2. Forest plots of continuous variables**

**
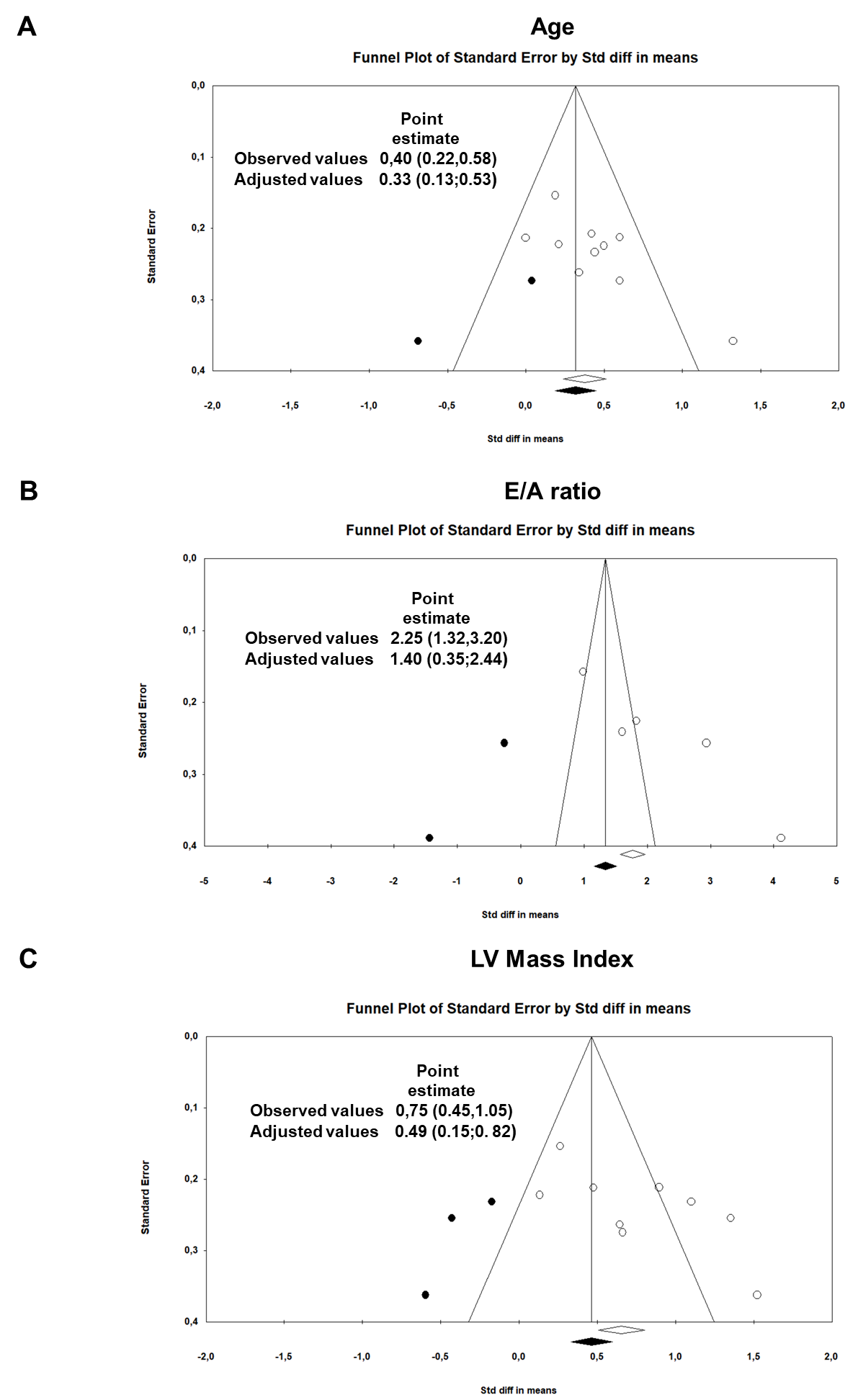
**

**Supplementary Figure S3.** **Funnel plots of effect size versus standard error for the studies evaluating Age (A), E/A ratio (B), and left ventricular mass index (C) in AS patients with and without cardiac amyloidosis.**

**
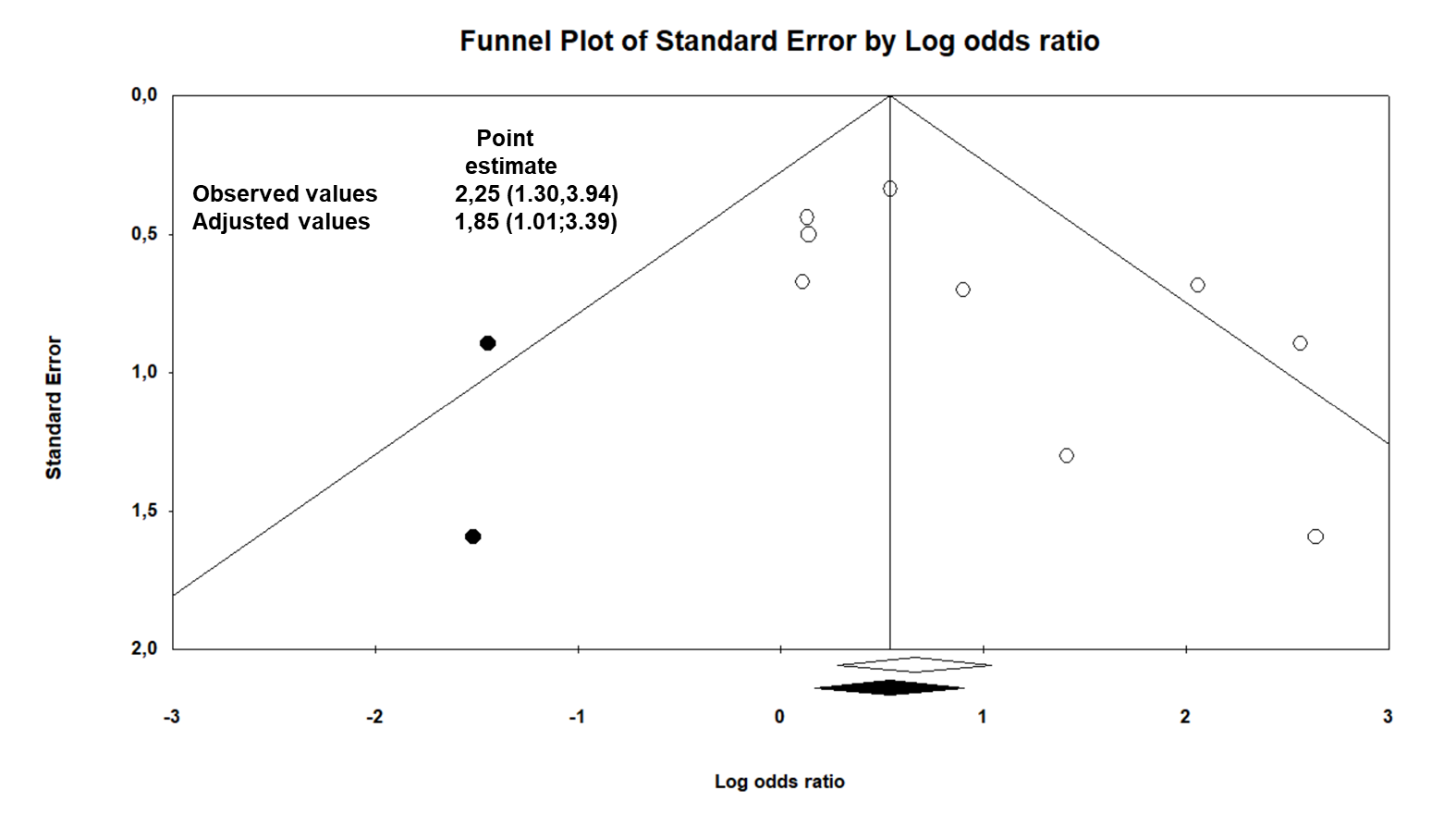
**

**Supplementary Figure S4.** **Funnel plots of effect size versus standard error for the studies evaluating overall mortality in AS patients with and without cardiac amyloidosis.**
